# Combating the Pandemic COVID-19: Clinical Trials, Therapies and Perspectives

**DOI:** 10.3389/fmolb.2020.606393

**Published:** 2020-11-17

**Authors:** Sabna Kotta, Hibah Mubarak Aldawsari, Shaimaa M. Badr-Eldin, Nabil Abdulhafiz Alhakamy, Shadab Md, Anroop B. Nair, Pran Kishore Deb

**Affiliations:** ^1^Department of Pharmaceutics, Faculty of Pharmacy, King Abdulaziz University, Jeddah, Saudi Arabia; ^2^Department of Pharmaceutics and Industrial Pharmacy, Cairo University, Cairo, Egypt; ^3^Department of Pharmaceutical Sciences, College of Clinical Pharmacy, King Faisal University, Al-Ahsa, Saudi Arabia; ^4^Department of Pharmaceutical Sciences, Faculty of Pharmacy, Philadelphia University, Amman, Jordan

**Keywords:** COVID-19, SARS-CoV-2, vaccine, convalescent plasma therapy, drug repurposing

## Abstract

The coronavirus disease-19 (COVID-19) is caused due to the infection by a unique single stranded enveloped RNA virus, severe acute respiratory syndrome coronavirus-2 (SARS-CoV-2). The COVID-19 has claimed many lives around the globe, and a promising solution to end this pandemic is still awaited. Till date neither an exact antiviral drug nor a vaccine is available in the market for public use to cure or control this pandemic. Repurposed drugs and supportive measures are the only available treatment options. This systematic review focuses on different treatment strategies based on various clinical studies. The review discusses all the current treatment plans and probable future strategies obtained as a result of a systematic search in PubMed and Science Direct database. All the possible options for the treatment as well as prophylaxis of COVID-19 are discussed. Apart from this, the article provides details on the clinical trials related to COVID-19, which are registered under ClinicalTrials.gov. Potential of drugs based on the previous researches on SARS-CoV, MERS-CoV, Ebola, influenza, etc. which fall under the same category of coronavirus are also emphasized. Information on cell-based and immunology-based approaches is also provided. In addition, miscellaneous therapeutic approaches and adjunctive therapies are discussed. The drug repurposing options, as evidenced from various *in vitro* and *in silico* models, are also covered including the possible future solutions to this pandemic.

## Introduction

Coronavirus disease-19 (COVID-19) is a rapidly transmitted respiratory disease that has recently attracted the worldwide public health attention since its declaration as a pandemic by the World Health Organization (WHO) on March 11, 2020 (WHO, 2020). Severe acute respiratory syndrome coronavirus-2 (SARS-CoV-2), the novel coronavirus responsible for COVID-19, primarily attacks the human respiratory system. Several upsurges of coronaviruses have previously occurred, like the severe acute respiratory syndrome (SARS) and the Middle East respiratory syndrome (MERS); both syndromes were considered as significant public health warnings (De Wit et al., 2016). In December 2019, some patients in China were diagnosed with pneumonia of an undetermined underlying cause (Bogoch et al., 2020; Lu H. et al., 2020). Early reports anticipated the start of a new coronavirus upsurge that was named by the WHO as COVID-19, on February 11, 2020. The high rate of human-to-human transmission of COVID-19 infection resulted in the necessity of patients’ isolation and a great urge for social distancing (Rothan and Byrareddy, 2020; Zhao et al., 2020).

Severe acute respiratory syndrome coronavirus-2 is the most recently discovered species of the coronaviruses (CoV) that infects humans, and it is categorized as a new strain of beta CoV. The genetics of the virus revealed above 80% similarity to SARS-CoV and above 50% to the MERS-CoV that originated in bats (Lu R. et al., 2020; Nadeem et al., 2020). Studies have been conducted to find a reservoir host or intermediate transmitter of the newly emerging virus. Early reports assume that two snake species as major reservoir of COVID-19. But, no evidences have been confirmed for animals other than bats and mammals as coronavirus reservoirs (Bassetti et al., 2020; Ji et al., 2020). Human-to-human transmission has been regarded as a likely mode of COVID-19 infection based on disease spreading within families and among people who were not exposed to animals (Graham Carlos et al., 2020; Helmy et al., 2020). Human-to-human transmission has been suggested to happen by unmediated contact or via droplets that outspread by coughing or sneezing from a person infected with the virus. Accordingly, the WHO has advised for keeping a distance of 1.5–2 m between people to reduce the likelihood of infection by nose or mouth droplets. However, the possibility of virus conveyance by airborne droplets over a distance of 2 m has been suggested by recent studies (Setti et al., 2020; van Doremalen et al., 2020).

The commonly identified clinical symptoms of COVID-19 disease are fever, dry cough, shortness of breath and excessive tiredness. Minor signs comprise of headache, sputum production, diarrhea, and lymphopenia (Borah et al., 2020; Ren et al., 2020). The symptoms, as mentioned earlier, are mostly revealed after an incubation period of about five days (Li Q. et al., 2020). A chest computed tomography (CT) scan in people with COVID-19 presents the clinical features of pneumonia (Huang et al., 2020). However, there are additional abnormal manifestations, including acute respiratory distress, severe cardiac side effects, and the presence of ground glass like opacities were found in sub-pleural areas of lungs in many cases. These opacities are likely to cause systemic and localized immune reactions that result in an elevated inflammatory response. Sorrowfully, the treatment of some cases with interferon did not exhibit promising clinical effects. Instead, the pulmonary opacities showed progression with consequent worsening of the condition (Dong D. et al., 2020; Lei et al., 2020). Recently it was found that the disease exhibits a broad spectrum of clinical signs and symptoms. Various case studies reported the involvement of all the vital organs of the body including heart, lungs, GIT, liver, kidneys, and central nervous system (CNS). In severe cases multisystem involvement can be seen and might lead to worse clinical outcomes as well as increased mortality (Gavriatopoulou et al., 2020).

No exact antiviral drug or vaccine against COVID-19 has been discovered yet. The only available option, other than using symptom alleviating agents, is utilizing broad-spectrum antiviral drugs comprising protease inhibitors and nucleoside analogs in an attempt to attenuate the viral infection (Lu, 2020). Amongst the antivirals that are recently recorded as having some effects against COVID-19 are oseltamivir (anti-flu drug), lopinavir/ritonavir (anti-human immunodeficiency virus; anti-HIV), and ganciclovir (Costanzo et al., 2020). High efficacy of the broad-spectrum antiviral remdesivir, which has been utilized for controlling the Ebola virus and the antimalarial agents like chloroquine and hydroxychloroquine, are also reported for controlling COVID-19 infection (Kumar et al., 2020; Ledford, 2020). Moreover, there are so many other molecules that are under development and testing. This article aims at reviewing the present and possible therapeutic options for the management of this emerging and widely spreading pandemic.

## Method

We systematically searched the researches, reviews, and case reports in PubMed and Science Direct database, using the keywords COVID-19 and treatment, and selected the relevant articles. Some of the cross-references were also accessed. After selecting the potential agents for the COVID-19 treatment, again a search was conducted using the drug name AND COVID. The search is refined by the term “2020.” The paper includes all the possible treatment strategies as well as all clinical trials for treatment or prevention of COVID-19 which are registered in NIH ClinicalTrials.gov.

## Mechanism of COVID-19 Infection and Possible Drug Targets

**FIGURE 1:**
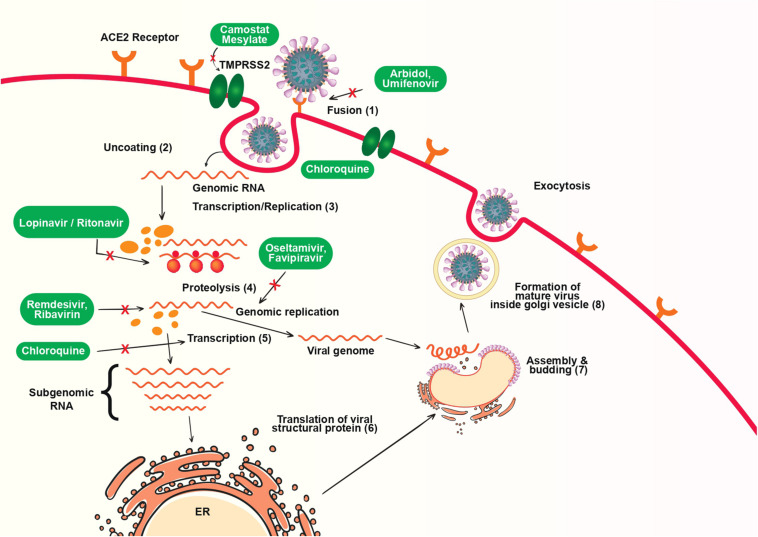
Replication of SARS-CoV-2 and possible drug targets.

The spike glycoprotein on the viral envelope is a major determinant of entry of virus to host cells by binding with its cellular receptor, angiotensin converting enzyme 2 (ACE-2). The viral infectivity and fusion are due to a significant proteolytic cleavage episode, and through clathrin-dependent as well as independent endocytosis. Once entered into the cells, the viruses release RNA which will synthesize two polyproteins and structural proteins, and starts replication. Then, the formation of nucleocapsid takes place by the combination of genomic RNA and nucleocapsid protein. Finally, the vesicles with viral particles combine with plasma membrane and discharge the viruses (Li X. et al., 2020). Different therapeutic agents against COVID-19 target at one or more different stages of the replication cycle. The mechanism of replication COVID-19 inside host cell and possible drug targets are shown in Figure 1.

**FIGURE 2:**
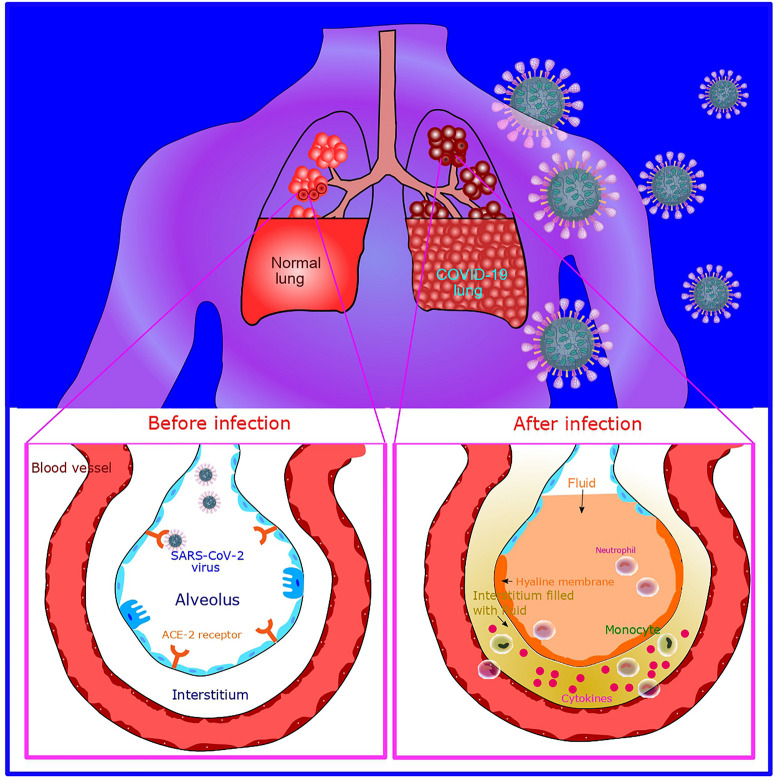
Illustration of COVID-19 infection and its effect on the lungs. After SARS-CoV-2 infection, macrophages are released which subsequently causes cytokine release (cytokine storm). Further recruitment of neutrophills results in the release of reactive oxygen species (ROS) for destroying the infected cells. Further, fluid filling into the interstitial space and alveoli occurs.

Various studies reported moderate to severe “cytokine storms” in severe patients. The “cytokine storm” which in turn leads to acute respiratory distress syndrome (ARDS), occurs due to the effects of combined action of several immunoactive molecules (Coperchini et al., 2020). This seems to be one of the most hazardous and life-threatening episodes in COVID-19. After binding to alveolar epithelial cells, the virus activates innate as well as the adaptive immune system. Following the SARS-CoV-2 infection macrophages are released as a response to inflammatory signals by type 2 cells. Cytokines are released by macrophages that in turn results in the release of more immune cells to the injury site. Cytokines cause vasodilatation also. Fluid accumulation in alveoli causes the damage of surfactant, and thus alveolar collapse which in turn affects the gas exchange. Further recruitment of neutrophils results in the release of reactive oxygen species (ROS) for destroying the infected cells. Also, the extensive release of cytokines occurs including interleukin-6 (IL-6), resulting in subsequent increase in the vascular permeability. This further leads to the entry of a large number of blood cells and fluid into the lungs, and causes dyspnea and respiratory failure (Zhang C. et al., 2020). The hyper-inflammation and cytokine storm syndrome is responsible ARDS and multi-organ failure. An illustration showing the effect of COVID-19 infection on the lungs is shown in Figure 2.

## Current Treatment Strategies

**FIGURE 3:**
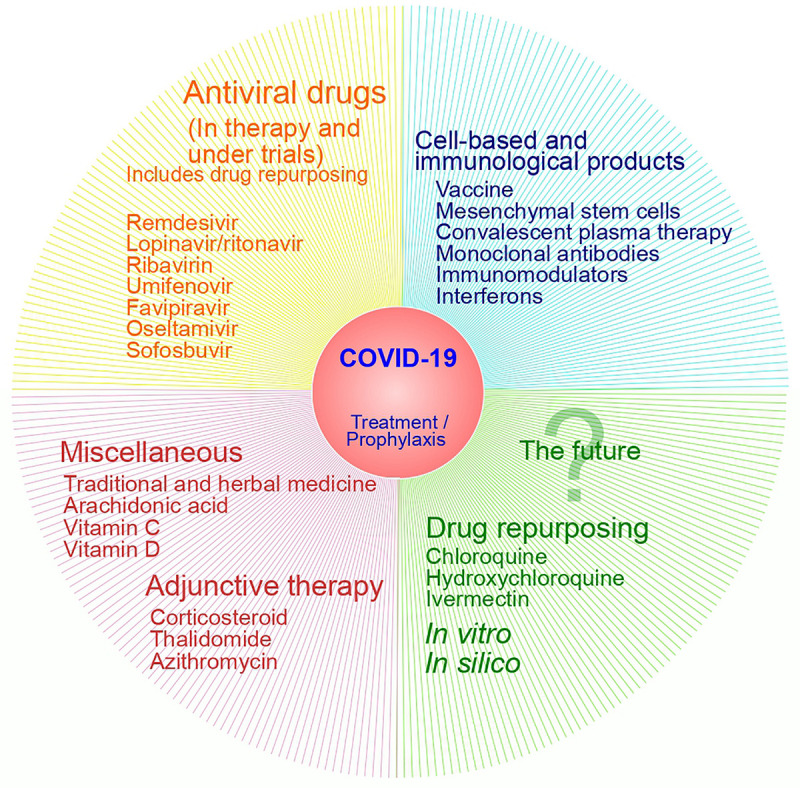
Therapeutic approaches against COVID-19.

Identifying effective therapeutic agents to fight this pandemic is urgently needed in this scenario (Li H. et al., 2020). A simplified classification of therapeutic options against COVID-19 is presented in Figure 3.

Drug repurposing is the only fastest approach to find potential candidates as a preventive or therapeutic measure for this new deadly pandemic (Ekins et al., 2020). Trials with ivermectin and hydroxychloroquine are examples of such an approach. At the same time, a large number of current researches are based on the testing of proven antiviral agents for related infections caused by SARS-CoV as well as MERS-CoV. The latter approach of the use of existing antiviral agents with proven efficiency against coronaviruses seems to be more promising. This argument could be justified by the fact that SARS-CoV-2 also belong to the same class of beta coronaviruses like SARS-CoV and MERS-CoV. Details of clinical trials on drug candidates against COVID-19 are presented in Table 1.

**TABLE 1:** Details of clinical trials on drug candidates against COVID-19.

	Intervention/treatment	Study type/Phase	Primary purpose	Number of participants	Sponsor (ClinicalTrials.gov Identifier)
1	Remdesivir	Interventional	Treatment	453	Capital Medical University (NCT04257656)
2	Remdesivir	Interventional	Treatment	308	(Capital Medical University) NCT04252664
3	Remdesivir	Expanded Access	Treatment		U.S. Army Medical Research and Development Command (NCT04302766)
4	Remdesivir	Interventional	Treatment	400	Gilead Sciences (NCT04292899)
5	Remdesivir	Interventional	Treatment	600	Gilead Sciences (NCT04292730)
6	Remdesivir	Expanded Access	Treatment	-	Gilead Sciences (NCT04323761)
7	Remdesivir	Interventional	Treatment	440	National Institute of Allergy and Infectious Diseases (NCT04280705)
8	Hydroxychloroquine	Interventional Phase 3	Treatment	440	Medical University of Vienna (NCT04336748)
9	Hydroxychloroquine Remdesivir	Interventional	Treatment	700	Oslo University Hospital (NCT04321616)
10	Chloroquine/hydroxychloroquine	Interventional Phase 2	Prevention	55000	Washington University School of Medicine (NCT04333732)
11	Chloroquine phosphate	Interventional	Treatment	250	Oxford University Clinical Research Unit, Vietnam (NCT04328493)
12	Chloroquine	Interventional Phase 2, Phase 3	Treatment	210	HaEmek Medical Center, Israel (NCT04333628)
13	Chloroquine phosphate	Interventional Phase 3	Treatment	400	Wrocław Medical University
					(NCT04331600)
14	Chloroquine or Hydroxychloroquine	Interventional	Prevention	4000	University of Oxford (NCT04303507)
15	Chloroquine analog, Nivolumab, Tocilizumab	Interventional Phase 2	Treatment	273	Centre Leon Berard (NCT04333914)
16	Chloroquine Diphosphate	Interventional Phase 2	Treatment	440	Fundação de Medicina Tropical Dr. Heitor Vieira Dourado (NCT04323527)
17	Azithromycin and Chloroquine	Interventional Phase 3	Treatment	1500	Population Health Research Institute (NCT04324463)
18	Azithromycin, Hydroxychloroquine	Interventional Phase 4	Treatment	226	Chronic Obstructive Pulmonary Disease Trial Network, Denmark (NCT04322396)
19	Darunavir and hydroxychloroquine	Interventional Phase 3	Treatment	3040	Fundacio Lluita Contra la SIDA (NCT04304053)
20	Hydroxychloroquine	Interventional Phase 3	Treatment	2486	Gangnam Severance Hospital (NCT04330144)
21	Hydroxychloroquine Sulfate	Interventional	Treatment	220	University Hospital, Akershus (NCT04316377)
22	Hydroxychloroquine azithromycin	Interventional Phase 3	Treatment	440	Hospital Israelita Albert Einstein (NCT04321278)
23	Hydroxychloroquine	Interventional	Treatment	1300	University Hospital, Angers (NCT04325893)
24	Levamisole, Budesonide, Formoterol, Lopinavir/Ritonavir hydroxychloroquine	Interventional Phase2 and 3	Treatment	30	Fasa University of Medical Sciences (NCT04331470)
25	Carrimycin lopinavir/ritonavir Arbidol, chloroquine phosphate	Interventional Phase 4	Treatment	520	Beijing YouAn Hospital (NCT04286503)
26	Oseltamivir, Hydroxychloroquine LopipinavirRitonavir, Darunavir Favipiravir	Interventional Phase 3	Treatment	80	Rajavithi Hospital (NCT04303299)
27	Favipiravir	Interventional	Treatment	210	Peking University First Hospital (NCT04333589)
28	Favipiravir	Interventional Phase 3	Treatment	100	Giuliano Rizzardini (NCT04336904)
29	Arbidol	Interventional Phase 4	Treatment	380	Jieming QU (NCT04260594)
30	ASC09(novel investigational protease inhibitor) lopinavir/ritonavir	Interventional	Treatment	160	First Affiliated Hospital of Zhejiang University (NCT04261907)
31	Lopinavir/ritonavir Hydroxychloroquine sulfate	Interventional phase 2	Treatment	150	Asan Medical Center (NCT04307693)
32	Lopinavir/ritonavir	Interventional phase 2	Treatment	440	Sunnybrook Health Sciences Centre (NCT04330690)
33	Hydroxychloroquine Lopinavir/ritonavir	Interventional Phase 3	Treatment	1200	Centre Hospitalier Universitaire de Saint Etienne (NCT04328285)
34	Hydroxychloroquine Oseltamivir Azithromycin	Interventional Phase 3	Treatment	500	Shehnoor Azhar (NCT04338698)
35	lopinavir/ritonavir Hydroxychloroquine Sulfate Losartan	Interventional Phase 2,3	Treatment	4000	Bassett Healthcare (NCT04328012)
36	Abidol hydrochloride, Oseltamivir Lopinavir/ritonavir	Interventional Phase 4	Treatment	400	Tongji Hospital
					(NCT04255017)
37	Lopinavir/ritonavir tablets Xiyanping injection	Interventional	Treatment	80	Jiangxi Qingfeng Pharmaceutical Co. Ltd. (NCT04295551)
38	lopinavir/ritonavir and Traditional Chinese Medicines	Interventional	Treatment	150	Beijing 302 Hospital (NCT04251871)
39	Methylprednisolone	Interventional Phase 2	Treatment	104	University of Trieste (NCT04323592)
40	Colchicine	Interventional Phase 3	Treatment	2500	Estudios Clínicos Latino América (NCT04328480)
41	Angiotensin 1-7	Interventional Phase 2/3	Treatment	60	Erasme University Hospital(NCT04332666)
42	Thalidomide	Interventional Phase 2	Treatment	40	First Affiliated Hospital of Wenzhou Medical University (NCT04273581)
43	Thalidomide	Interventional Phase 2	Treatment	100	First Affiliated Hospital of Wenzhou Medical University NCT04273529
44	Dietary Supplement: Natural Honey	Interventional Phase 3	Treatment	1000	Misr University for Science and Technology (NCT04324489)

### Antiviral Agents

#### Remdesivir

Remdesivir is an investigational antiviral agent and also the first drug under clinical trial in the United States as an experimental cure for COVID-19 (Holshue et al., 2020). It possesses broad spectrum of action against different RNA viruses, such as MERS-CoV. Remdesivir and chloroquine are shown to be very efficient in the control of novel coronavirus infection *in vitro* (Li Q. et al., 2020). To understand the mechanism of inhibition, in a recent study, the MERS-CoV non-structural proteins were co-expressed in insect cells as a part of the polyprotein. The research demonstrated that remdesivir acts by inhibiting the nucleotide analog of RNA-dependent RNA polymerase (Gordon et al., 2020). An investigation in rhesus macaque model infection of MERS-CoV on preventive as well as the therapeutic potential of remdesivir revealed that the drug could reduce damage to the lungs and inhibit virus replication when administered either previous to or following infection (De Wit et al., 2020). The active metabolite of remdesivir can interact with both the active sites of enzyme and can produce delayed chain termination as well as distorted excision due to the ribose 1’-CN group, which is responsible for the enhanced antiviral action than other existing analogs (Shannon et al., 2020). Nine out of the ten ongoing clinical trials aim to evaluate the antiviral activity of remdesivir in SARS-Cov-2 infection. One of the studies is purely for the assessment of the adverse effects of the drug.

#### Umifenovir (Arbidol^®^)

Umifenovir is an antiviral agent that acts through multiple pathways and is effective against a variety of enveloped as well as non-enveloped DNA and RNA viruses. It is used for prophylaxis as well as treatment of influenza. It has been in use for more than 25 years in Russia and 14 years in China (Blaising et al., 2014). Since *in vitro* studies proved the efficacy of this agent in SARS, it is being now used in the empirical therapy of COVID-19 in China (Song et al., 2020; Zhang J. et al., 2020). This drug is given orally for a maximum of 10 days at a dose of 200 mg, three times per day (Dong L. et al., 2020). In a study conducted in China, four patients administered with lopinavir/ritonavir, umifenovir, and one with traditional Chinese medicine along with essential support care. Three of them gained considerable relief from pneumonia, and two showed a negative viral test after the treatment period. The last patient with severe respiratory problems also showed significant improvement with this treatment procedure (Wang et al., 2020a). In a retrospective cohort study, 75% of the patients who took a combination of oral umifenovir and lopinavir/ritonavir recovered in 7 days as compared to 35% of patients who received lopinavir/ritonavir alone. And after 14 days, the viral clearance was achieved in 94% of the patients who received the drug combination, but it was only 69% in the other group (Deng et al., 2020). Also, umifenovir treatment could increase the discharging rate as well as a decline in the mortality rate(Wang et al., 2020b). Four trials are registered in ClinicalTrials.gov on umifenovir for COVID-19.

#### Lopinavir and Ritonavir

Both of these drugs are antiretroviral protease inhibitors and co-administration of these drugs improves the pharmacokinetics of both. Ritonavir is a potent inhibitor of microsomal enzyme cytochrome P-450 3A4, so co-administration of ritonavir leads to the increased bioavailability and half-life of the co-administered lopinavir (Cooper et al., 2003). Lopinavir/ritonavir is given two times per day in a dose of 400 mg/100 mg (Dong L. et al., 2020). After the lopinavir/ritonavir administration, coronavirus titers were null in a 54 years old male patient in Korea (Lim et al., 2020). In India, the Central Drugs Standard Control Organization agreed to use lopinavir/ritonavir therapy for 14 days with informed consent in high-risk categories who are symptomatic COVID-19 patients (Bhatnagar et al., 2020). Nevertheless, no benefit was observed in adult patients admitted in the hospital due to severe COVID-19 with lopinavir/ritonavir therapy beyond standard care (Cao et al., 2020). More similar remarks were noticed and accordingly, the benefits of this combination are still doubtful (Kupferschmidt and Cohen, 2020). As a result, experts opined that the effectiveness of remdesivir and lopinavir/ritonavir should be confirmed by a randomized controlled trial. A retrospective data of pediatric patients with confirmed COVID-19 shows that all 36 children received interferon-alfa (INFα), 14 received lopinavir/ritonavir, and 6 needed oxygen inhalation resulted in full recovery irrespective of the drug within 14 ± 3 days. Treatment with INFα along with lopinavir/ritonavir plus ribavirin showed a beneficial action in COVID-19 therapy (Yuan et al., 2020). At the same time, some reports expressed that the use of lopinavir/ritonavir along with adjuvant drugs should be encouraged for the treatment of patients with COVID-19 (Ye et al., 2020). More than 20 clinical trials are registered for the evaluation of this combination in COVID-19.

#### Favipiravir

Favipiravir, a derivative of pyrazine carboxamide, is a purine nucleic acid analog that interferes with the replication of the virus and inhibits RNA dependent RNA polymerase of RNA viruses. It possesses broad-spectrum antiviral activity and is effective against the influenza virus, bunyavirus, arenavirus, and filovirus (Du and Chen, 2020; Singh et al., 2020). An early result of a clinical trial reveals that favipiravir has more strong anti-viral activity than lopinavir/ritonavir with significantly less adverse effects (Chavez et al., in press; Zhai et al., 2020). Due to its efficacy on virus clearance, the Turkish ministry of health approved favipiravir for treating critical patients with Covid-19 pneumonia (Kodaz, 2020). Eleven clinical trials are registered for this drug in COVID-19.

#### Oseltamivir

It is a neuraminidase inhibitor that hinders the neuraminidase enzyme expressed on the surface of the virus. This enzyme is needed for the release of the virus from the cells. It is approved for the prophylaxis as well as for the treatment of influenza (Li et al., 2012). Five trials are registered for this drug in COVID-19 treatment and details are given in Table 1.

In the study by Ding et al. about the clinical characteristics of COVID-19 patients, all the 115 subjects received oseltamivir along with antibiotics and oxygen inhalation and all recovered without the need for intensive care unit (ICU; Ding et al., 2020). A 71-year-old woman tested positive for COVID-19 with a childhood history of psoriasis pointed out exacerbation of psoriasis after oseltamivir and hydroxychloroquine treatment. Hydroxychloroquine inhibits epidermal transglutaminase, which leads to the collection of the epidermal cells, and to date, there were no reports that oseltamivir may affect psoriasis. Thus, it could be reasonably argued that hydroxychloroquine may lead to a global increase in the number of psoriasis (Kutlu and Metin, 2020).

In the study conducted by Huang et al. (2020), all the 41 patients were given empirical antibiotic treatment, and 93% received oseltamivir also, but the results are yet to be known. A 43-year-old female patient was recovered and discharged from the hospital after the use of oseltamivir along with traditional Chinese medicine. Unfortunately, this patient showed a positive SARS-CoV-2 test again after 22 days of hospital discharge, however, convalescent plasma therapy (CPT) along with other measures made her condition better (Chen D. et al., 2020; Luo, 2020).

#### Ribavirin

Ribavirin is a guanosine analog and nucleoside inhibitor to stop viral RNA synthesis. It was widely used to treat SARS in combination with or without steroids in severe cases. Virtual screening of some FDA approved medicines against SARS-CoV-2 main protease (M^*pro*^) has been done and found that ribavirin, telbivudine, vitamin B12, and nicotinamide has an excellent docking score and can be made use in the treatment of COVID-19 (Kandeel and Al-Nazawi, 2020). In another molecular docking study, it was found that ribavirin, galidesivir, sofosbuvir, remdesivir, and tenofovir are effective agents in the treatment of COVID-19 since these drugs can tightly bind to the viral RNA dependent RNA polymerase (Elfiky, 2020). Since ribavirin shows the adverse effect of decreasing hemoglobin levels, it is not recommended for patients with respiratory distress (Jean et al., 2020). Out of the two clinical trials registered for this drug, one is completed, but results are not yet available.

### Cell-Based and Immunological Products

#### Monoclonal Antibodies

In COVID-19 infection, activation of a huge number of mononuclear macrophages and T lymphocytes occur which results in the production of cytokines such as IL-6. This IL-6 will bind to the IL-6 receptor on the target cells, which leads to cytokine storm as well as dangerous inflammatory responses in the lungs and other organs (Xu et al., 2020).

Tocilizumab, a humanized monoclonal antibody that acts as a blocker of the IL-6 receptor, can bind to the IL-6 receptor with high affinity. It can prevent IL-6 itself from binding to its receptor, making it unable to injure the target cells, and lessen the inflammatory responses (Zhang C. et al., 2020). Its use is considered as one of the latest treatment strategies against COVID-19 (Bersanelli, 2020). The results of a retrospective study in fifteen COVID-19 patients, including moderate to critically ill, suggests that tocilizumab can be an efficient management option for patients with a risk of cytokine storms (Luo et al., 2020). Treatment with Tocilizumab, which blocks IL-6 receptors, results in notable outcomes like reduction in the elevated body temperature and improved respiratory function (Fu et al., 2020). Tocilizumab is also referred to as a promising choice for the treatment of the hyperinflammatory state associated with this infection in the second edition of “Vademecum for the Treatment of People With COVID-19” (Lombardy Section Italian Society Infectious And Tropical Diseases, 2020). In a COVID-19 patient with multiple myeloma, the treatment with tocilizumab was successful. The study recommends the need for randomized clinical trials for detailed evidence (Zhang X. et al., 2020). Further, several studies have been reported the use of tocilizumab as a promising treatment option for COVID-19 related respiratory failure (Bachanova et al., 2020; Bennardo et al., 2020; Buonaguro et al., 2020; Cellina et al., 2020; Ceribelli et al., 2020; Liu B. et al., 2020; Michot et al., 2020; Mihai et al., 2020).

Coronavirus neutralizing antibodies mainly aim the spike proteins on the surface of the virus, which mediate the entry into host cells. Receptor binding can generate irreversible conformational alteration in the spike proteins and thus inhibits the viral fusion with host cells (Wang C. et al., 2020). It is now suggested that CR3022, a SARS-CoV-specific human monoclonal antibody, has the potential to emerge as an agent against SARS-CoV-2, along with other neutralizing antibodies (Tian et al., 2020). Sarilumab, gimsilumab, lenzilumab, etc. are the other monoclonal antibodies in the trial. More than 40 clinical trials on monoclonal antibodies are registered, including those on tocilizumab, sarilumab, gimsilumab, and lenzilumab. Details of selected clinical trials on biological agents against COVID-19 are provided in Table 2.

**TABLE 2:** Details of clinical trials on biological agents against COVID-19.

No.	Intervention/treatment	Study type/Phase	Primary purpose	No. of participants	Sponsors and Collaborators (ClinicalTrials.gov Identifier)
1	Lopinavir/ritonavir Hydroxychloroquine sulfate Baricitinib (Janus kinase inhibitor) Sarilumab (anti-IL-6 receptor)	Interventional (Phase 2)	Treatment	1000	Lisa Barrett, Nova Scotia Health Authority (NCT04321993)
2	Xiyanping injection Lopinavir/ritonavir, alpha-interferon nebulization	Interventional (Phase 2,3)	Treatment	348	Jiangxi Qingfeng Pharmaceutical Co. Ltd. (NCT04275388)
3	Remdesivir Lopinavir/ritonavir, Interferon Beta-1A, Hydroxychloroquine	Interventional	Treatment	3100	Institut National de la Santé Et de la Recherche Médicale, France (NCT04315948)
4	lopinavir/ritonavir, remdesivir, interferon beta-1a, chloroquine and/or azithromycin	Observational	Adverse events	1000	Groupe Hospitalier Pitie-Salpetriere (NCT04314817)
5	Hydrocortisone, Ceftriaxone, Moxifloxacin, Levofloxacin, Piperacillin-tazobactam, Ceftaroline, Amoxicillin, clavulanate, Macrolide, oseltamivir, Lopinavir/ritonavir Hydroxychloroquine Interferon-β1a Anakinra	Interventional	Treatment (Phase 4)	6800	MJM Bonten (NCT02735707)
6	Lopinavir/ritonavir, Ribavirin, Interferon Beta-1B	Interventional (Phase 2)	Treatment	70	The University of Hong Kong (NCT04276688)
7	ASC09F Oseltamivir Ritonavir Oseltamivir	Interventional (Phase 3)	Treatment	60	Tongji Hospital (NCT04261270)
8	Hydroxychloroquine Lopinavir/Ritonavir Interferon Beta-1A Interferon Beta-1B	Interventional (Phase 4)	Treatment	60	Shahid Beheshti University of Medical Sciences
9	Abidol Hydrochloride Interferon	Interventional (Phase 4)	Treatment	100	Tongji Hospital (NCT04254874)
10	Recombinant human interferon Alpha-1b thymosin alpha 1	Interventional (Phase 3)	prevention	2944	Shanghai Jiao Tong University School of Medicine (NCT04320238)
11	Ganovo, ritonavir, Interferon	Interventional (Phase 4)	Treatment	11	The Ninth Hospital of Nanchang (NCT04291729)
12	Recombinant human interferon α1β	Interventional (early Phase 1)	Treatment	328	Tongji Hospital (NCT04293887)
13	Bromhexine Hydrochloride, Arbidol Hydrochloride Recombinant Human Interferon α2b	Interventional	Treatment	60	Second Affiliated Hospital of Wenzhou Medical University (NCT04273763)
14	Emapalumab(Anti-interferon Gamma) Anakinra (Interleukin-1Receptor Antagonist)	Interventional (Phase 2/3)	Treatment	54	Swedish Orphan Biovitrum (NCT04324021)
15	Tocilizumab	Observational	Treatment	30	University of L’Aquila (NCT04332913)
16	Favipiravir Tocilizumab	Interventional	Treatment	150	Peking University First Hospital (NCT04310228)
17	INO-4800, a Prophylactic Vaccine	Interventional (non-randomized)	Prevention	40	Inovio Pharmaceuticals (NCT04336410)
18	Biological: UC-MSCs	Interventional	Prevention	10	ZhiYong Peng (NCT0426952)
19	Biological: ChAdOx1 nCoV-19	Interventional (Phase 1/2)	Treatment	510	University of Oxford (NCT04324606)
20	Tocilizumab Sarilumab	Interventional (Phase 2)	Treatment	200	Marius Henriksen (NCT04322773)
21	Siltuximab Methylprednisolone	Interventional (Phase 2)	Treatment	100	Judit Pich Martínez (NCT04329650)
22	Tocilizumab Pembrolizumab (MK-3475)	Interventional (Phase 2)	Treatment	24	MedSIR (NCT04335305)

#### Interferons

Interferons are signaling proteins and have antiviral activity. Viruses trigger the release of interferons by the host cells. Type 1 interferons possess a wide range of antiviral effects *in vitro* and a recent clinical trial proved its efficacy in the treatment of MERS-CoV. With regard to this, interferon wastried in clinical trials as a treatment option for COVID-19. The β subtype is found more promising, and the treatment in the early stages of the infection is recommended (Sallard et al., 2020). The combination of ribavirin and INFα has been the most commonly used therapy to treat MERS outbreaks in South Korea (Kim et al., 2016). Due to the effectiveness of this therapy, this combination is recommended to treat COVID-19 infection in the fifth edition of the National Health Commission’s Regimen of China (Du et al., 2020).

#### Mesenchymal Stem Cells

The human umbilical cord mesenchymal stem cell (MSC) possesses outstanding immunomodulatory and strong anti-inflammatory functions with proper safety (Metcalfe, 2020). Liang et al. (2020) reported that treatment with allogeneic human umbilical cord MSCs in a 65-year-old critically ill female patient with COVID-19 showed a significant and positive outcome with good tolerance. So this kind of therapy is an ideal choice for the management of seriously ill COVID-19 patients (Liang et al., 2020). In another study, seven patients who are dangerously ill with COVID-19, MSCs therapy significantly improved their condition without any adverse effects (Leng et al., 2020). Therefore, MSC therapy is a safe and effective option for critical cases of pneumonia associated with COVID-19. Even though the results are promising, proper clinical investigations are required for these kinds of cell-based therapies (Khoury et al., 2020). A total of 29 clinical trials are registered for the efficacy and safety evaluation of MSCs in COVID-19.

#### Convalescent Plasma Therapy

**FIGURE 4:**
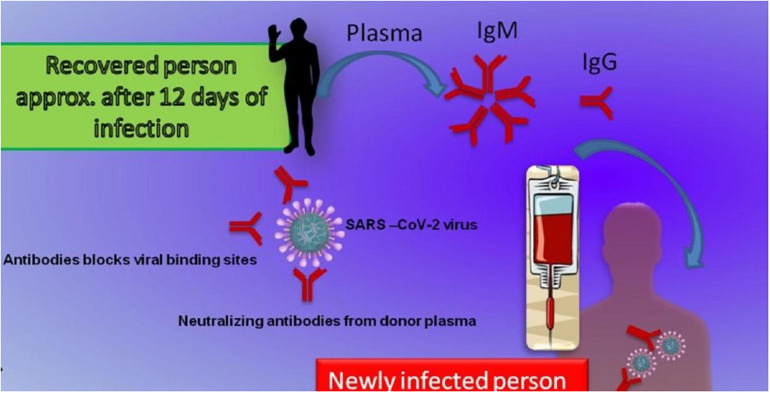
Schematic representation of the convalescent plasma therapy along with its mechanisms of action. A person recovered from COVID-19 infection produces a sufficient amount of specific antibodies in 12–14 days. The plasma with neutralizing antibodies mainly IgM and IgG can be transferred to produce immediate immunity in suspected or infected persons. IgG and IgM anti-SARS-CoV-2 antibodies will bind to specific sites and neutralize the virus.

Convalescent plasma therapy is considered one of the advanced options sought for the treatment of COVID-19 (Zhao and He, 2020). Convalescent or immune plasma is the plasma collected from individuals who are cleared of infection with a sufficient amount of developed and antibodies. Convalescent plasma therapy will help to get immediate immunity for a short time in susceptible individuals (Bloch et al., 2020). Neutralizing antibodies are critical in virus clearance and vital in defense against various viral diseases. Passive immunity achieved due to convalescent plasma can provide neutralizing antibodies that can control the infection. In addition to antiviral action, convalescent plasma can also cause immunomodulation. Some antibodies can cause the inhibition of complement cascade and thus can control the formation of immune complexes. Convalescent plasma has anti-inflammatory effects also due to the action of a network of autoantibodies and can manage an overactive immune system. The effectiveness of this therapy is highly related to the concentration of neutralizing antibodies in the collected plasma (Rojas et al., 2020). A brief mechanism of CPT is depicted in Figure 4.

Despite this apparent advantage, several challenges are also associated with CPT such as anaphylactic reactions, transfusion transmitted infections, transfusion associated acute lung injury, circulatory overload, hemolysis, etc. Owing to the importance of this approach, the possibilities and challenges of CPT are now well discussed and described (Roback and Guarner, 2020).

From the previous studies related to SARS, it was reported that CPTresulted in a shorter hospital stay period as well as a lower death rate as compared to the control group (Soo et al., 2004; Cheng et al., 2005; Chen L. et al., 2020). Five seriously sick patients with COVID-19 showed promising results when received plasma transfusion. After the transfusion, body temperature and viral loads were declined and turned to negative within 12 days (Shen et al., 2020). In another study, a single dose of 200 mL of convalescent plasma antibody titers over 1:640 was transfused to 10 patients along with antiviral agents and supportive measures. The results confirmed that this therapy was well tolerated and could be able to neutralize viremia in critical COVID-19 cases (Duan et al., 2020).

In April 2020, the US FDA permitted CPT in dangerously sick patients with COVID-19 (Tanne, 2020). Two COVID-19 patients with severe pneumonia and ARDS showed a positive result by CPT along with corticosteroids (Ahn et al., 2020). A good number of clinical trials are registered for studying the safety and efficacy evaluation of CPT in COVID-19 patients (Table 3). Many clinical results showed that CPT produces remarkable improvement in clinical symptoms as well as radiological and biochemical parameters related with the SARS-CoV-2 infection (Bakhtawar et al., 2020).

**TABLE 3:** Details of convalescent plasma therapies at clinical trials.

No.	Intervention/treatment	Study type/Phase	Primary purpose	Number of participants	Sponsors and Collaborators (ClinicalTrials.gov Identifier)
1	Convalescent Plasma	Interventional (Phase 1)	Treatment	20	Hospital San Jose Tec de Monterrey (NCT04333355)
2	Anti-SARS-CoV-2 convalescent plasma	Interventional (Early Phase 1)	Treatment	20	Orthosera Kft. (NCT04345679)
3	Convalescent Plasma Transfusion	Interventional (Phase 2)	Treatment	20	Institute of Liver and Biliary Sciences, India (NCT04346446)
4	Convalescent Plasma	Interventional (Phase 2)	Treatment	15	Saint Francis Care (NCT04343261
5	COVID-19 convalescent plasma	Expanded Access	Treatment	Expanded Access	Mayo Clinic (NCT04338360)
6	Transfusion of COVID-19 convalescent plasma	Interventional (Phase 2)	Treatment	120	Assistance Publique - Hôpitaux de Paris (NCT04345991)
7	convalescent plasma from recovered COVID 19 donor	Interventional (Phase 2)	Treatment	40	King Fahad Specialist Hospital Dammam (NCT04347681)
8	Convalescent Plasma	Interventional (Phase 1/2)	Treatment	500	Stony Brook University (NCT04344535)
	Standard Donor Plasma				
9	Convalescent Plasma	Interventional (Phase 2)	Treatment	55	Hackensack Meridian Health (NCT04343755)
10	Convalescent Plasma	Interventional (Phase 2)	Treatment	426	Erasmus Medical Center (NCT04342182)
11	Convalescent Plasma	Interventional (Phase 2)	Treatment	278	Cristina Avendaño Solá (NCT04345523)
12	Convalescent Plasma	Interventional (EarlyPhase1)	Treatment	10	University of Chicago (NCT04340050)
13	Plasma Hydroxychloroquine	Interventional (Phase 1/2)	Treatment	80	Universidad del Rosario (NCT04332835)
	Azithromycin				
14	Convalescent Plasma	Interventional	Treatment	30	Mazandaran University of Medical Sciences (NCT04327349)
15	Convalescent Plasma	Observational		15	Shanghai Public Health Clinical Center (NCT04292340)
16	Anti- SARS-CoV-2 Plasma SARS-CoV-2 non-immune Plasma	Interventional (Phase 2)	Treatment	150	Johns Hopkins University (NCT04323800)
17	Convalescent anti-SARS-CoV-2 plasma Sarilumab, Baricitinib, Hydroxychloroquine	Interventional (Phase 3)	Treatment	1500	Thomas Benfield (NCT04345289)
18	high-titer anti-Sars-CoV-2 plasma, oxygen therapy	Interventional (Phase 1)	Treatment	115	Baylor Research Institute (NCT04333251)
19	Anti-coronavirus antibodies (immunoglobulins)obtained with DFPP from a convalescent patient	Interventional	Treatment	10	A.O. Ospedale Papa Giovanni XXIII (NCT04346589

#### Vaccines

**FIGURE 5:**
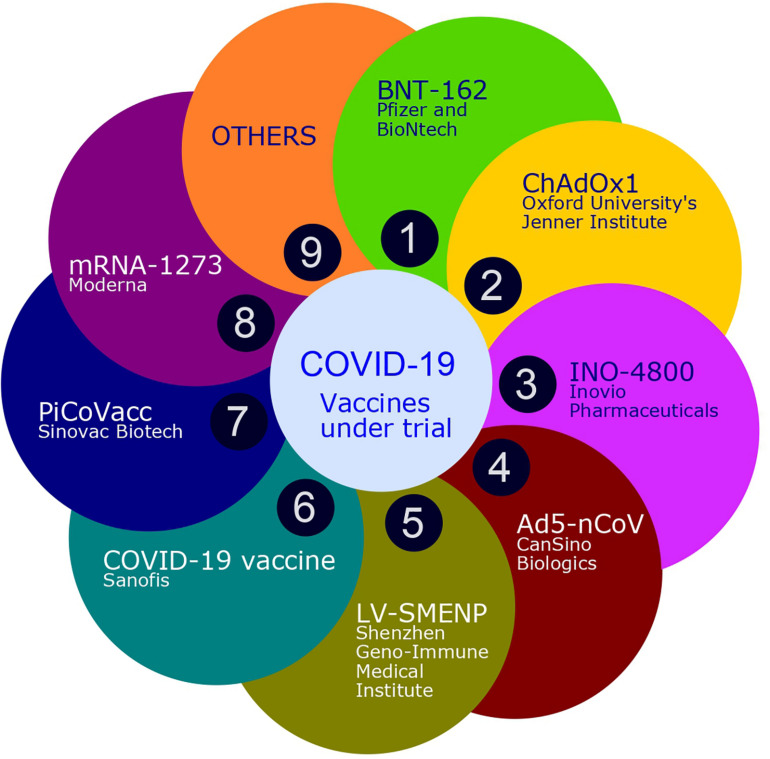
Some of the ongoing vaccine trials in lead which are specific to COVID-19.

Vaccine development needs many years to reach the market under normal circumstances. Fortunately, the data generated in the research on SARS-CoV as well as MERS-CoV helped in an express design and development of the COVID-19 vaccine. Thus, within three months of the emergence of COVID-19, a specific vaccine candidate entered Phase I clinical trials, and WHO announced the news of the availability of licensed vaccine for widespread use by the middle of 2021. Presently, different SARS-CoV and MERS-CoV vaccine candidate are in clinical trials against COVID-19. At the same time, thorough investigations are required urgently to study the risk of immune enhancement. Therefore, along with early clinical trials, clinical evidence is also needed to support the possibility of immune enhancement (de Alwis et al., 2020). According to the latest WHO report there are more than 169 COVID-19 vaccine candidates currently under clinical trials. Among these clinical candidates, 26 are in the various phase of human trials and 10 reached up to phase 3 of the clinical trial. Figure 5 shows some of the ongoing vaccine trials in lead which are specific to COVID-19.

### Miscellaneous/Adjunctive Therapy

#### Traditional Chinese Medicine

Various Traditional Chinese Medicines like Shuanghuanglian oral liquid and Lianhuaqingwen capsule were also tried for treating SARS-CoV-2 infection and found satisfactory outcome. Some of them, like YinHu QingWen, Fuzheng Huayu, are in registered clinical trials (Wang L. et al., 2020). A total of 8 clinical trials are registered for the evaluation of the effectiveness of Traditional Chinese Medicine.

#### Vitamins

An adequate level of vitamin D is required to suppress the adhesion molecule (CD26), which helps in invasion to host cells. Since, vitamin D deficiency leads to increased risk of respiratory infections, supplementation with adequate dose is highly recommended in high-risk categories (McCartney and Byrne, 2020). Clinical studies show that a large dose of vitamin C can prevent viral infections. Thus, early use of antioxidants like vitamin C in large doses may be helpful to fight COVID-19 (Cheng, 2020).

#### Corticosteroids

Even though corticosteroids are used as adjuvant therapy in COVID-19 infection, WHO and the United States Centre for Disease Control and Prevention recommend that it should not be regularly used in COVID-19 patients, except indicated for other conditions. Low to moderate doses with close monitoring as a short course may be beneficial. More than ten trials are registered for the evaluation or comparison of the safety as well as efficacy of different corticosteroids in COVID-19 (Rosa and Santos, 2020).

#### Thalidomide

The severe lung injury in COVID 19 may be related to the extreme immune response as a result of cytokine storm. Thalidomide is an immunomodulatory agent, and it can stimulate T cells, decrease TNF-α production, and enhance the secretion of interleukins and natural killer cells. Its anti-inflammatory role is due to the capacity to reduce TNF-α by enhancing the degradation of mRNA in blood cells. Two trials are registered to test the safety and effectiveness of thalidomide in treating moderate or severe COVID-19 (Newfield, 2018).

### Drug Repurposing Approach

#### Chloroquine or Hydroxychloroquine

Chloroquine is a popular drug used for the prophylaxis of malaria and amebiasis and also to treat autoimmune diseases like rheumatoid arthritis and lupus erythematosus. A wide range of mechanisms has been proposed for the action of chloroquine (Cortegiani et al., 2020). By altering the binding of the virus to the cell surface receptor, it can hold back the pre-entry step to the host cell (Vincent et al., 2005). Also, it can impair pH reliant endosome dependent entrance of enveloped viruses like chikungunya and dengue viruses (Gay et al., 2012; Wong and Chu, 2018). Post-translational variation of viral proteins can also be damaged by chloroquine. It can also spoil the viral protein maturation (Devaux et al., 2020). In addition, it acts as an immunomodulatory agent by the regulation of pro-inflammatory cytokines and cell signaling.

Several trials on the effectiveness of this agent have been found to be registered in the Chinese Clinical Trial Registry. Based on this, Liu and associates examined the antiviral action of hydroxychloroquine and chloroquine against COVID-19 *in vitro*. It was found that both the agents elevate the intracellular organelles’ pH, which is critical in membrane fusion. Both the drugs were able to exert their actions to inhibit the viral entry as well as at some post-entry stages. It was found that the drugs could block the transport of the virus from the early endosomes to endolysosomes, which is essential for the release of the viral genome. The study concludes that hydroxychloroquine is as effective as chloroquine for the *in vitro* inhibition of SARS-CoV-2 infection with comparatively less toxicity (Liu J. et al., 2020). A survey in a University Hospital in Marseille reassured the disappearance or decrease of viral load in hydroxychloroquine treated patients, and the result is reinforced by concomitant use of azithromycin (Gautret et al., 2020).

Wang and associates demonstrated the effectiveness of chloroquine at entry as well as post-entry phases of COVID-19 infection *in vitro* cell lines. Further, the immune modulation property can induce synergism in its antiviral activity *in vivo* (Wang M. et al., 2020). Still, there is an opinion that the option of using chloroquine for the treatment of COVID-19 should be properly analyzed in light of the new hopeful declarations, by considering the possible side effects (Colson et al., 2020; Touret and de Lamballerie, 2020). Chloroquine causes under expression of phosphatidylinositol binding clathrin assembly protein and thereby affects endocytosis (Hu et al., 2020). Meanwhile, the assessment of safety and efficiency of chloroquine for treating COVID-19 demands the need for safety data from urgent high-quality trials from various geographical areas (Cortegiani et al., 2020). Interestingly, chloroquine phosphate shortened the course of the disease in clinical trials and thus seems to be better than the control, for the inhibition of pneumonia (Gao et al., 2020) Also, *in vitro* study revealed that hydroxychloroquine shows the more powerful effect as compared to chloroquine (Yao et al., 2020). As of now, more than 40 clinical trials are registered to assess and/or compare the effectiveness and/or safety of chloroquine and hydroxychloroquine.

#### Metronidazole

Metronidazole is another nucleic acid synthesis inhibitor and a potential candidate which can counter most of the immunopathological symptoms of SARS-CoV-2 infection. *In vitro* as well as *in vivo* studies proved that this drug could reduce cytokine levels, which are generally increased during this disease. *In vitro* studies proved that metronidazole at high doses had a marked inhibitory effect on lymphoproliferative assay (Fararjeh et al., 2008; Rizzo et al., 2010). Also, it can reduce neutrophil-generated ROS in the event of inflammation. But studies with a big number of groups are needed to prove its effectiveness (Gharebaghi et al., 2020)

Sofosbuvir, an FDA approved nucleotide polymerase inhibitor mainly used for the management of hepatitis C is under test by a Chinese research foundation (Vellingiri et al., 2020). Previously sofosbuvir was used along with ribavirin and interferon. The use of sofosbuvir is reported in the management of the Zika virus also (Cheema et al., 2019).

#### Some Other Potential Agents

Baricitinib, carfilzomib, indinavir, baloxavir, ruxolitinib, fedratinib, and azvudine are some other potential agents to treat this respiratory disease (Peter et al., 2020). Among these, fedratinib, baricitinib, and ruxolitinib are potent anti-inflammatory agents and powerful Janus kinase inhibitors (Stebbing et al., 2020) which are approved for rheumatoid arthritis and myelofibrosis. It is reported that these drugs are effective to control the increased levels of cytokines usually observed in COVID-19 patients (Stebbing et al., 2020). Twelve clinical trials have been registered to evaluate the efficacy and safety of ruxolitinib in COVID-19 patients. Baricitinib is not considered an ideal choice for the treatment of COVID-19, since it may enhance the chance of co-infection and increase the incidence of anemia (Praveen et al., 2020). Eight clinical trials are registered for the evaluation of the efficacy of baricitinib in COVID-19 patients. The immune-modulating and anti-inflammatory agents are not usually recommended in pneumonia associated with COVID-19. Nevertheless, as per the pathology of pulmonary edema as well as the formation of hyaline membrane, it could be reasonably assumed that well-timed and appropriate therapy with immunomodulators along with other supportive measures may save the COVID-19 patients from ARDS. Based on these considerations, one clinical trial is going on for the evaluation of fingolimod, an immunology modulator generally used in multiple sclerosis (ClinicalTrails, 2020).

Nitazoxanide is an antiprotozoal drug with a broad-spectrum antiviral activity, which can also reduce the production of pro-inflammatory cytokines (Kelleni, 2020). With regard to this feature, it has been registered for studies in more than 6 clinical trials as monotherapy or as combination therapy for the management of COVID-19 (ClinicalTrails, 2020).

Severe acute respiratory syndrome coronavirus-2 infection depends on ACE2 and TMPRSS2 proteins on the host cell surface and these can be blocked by protease inhibitors (Nadeem et al., 2020). Camostat mesylate is a synthetic serine protease inhibitor that can inhibit TMPRSS2 protein in lung cells (Hoffmann et al., 2020; McKee et al., 2020). Nafamostat mesylate, another TMPRSS2 serine protease inhibitor, may also prevent the entry of virus into the host cell. Cell culture experiments proved that nafamostat mesylate inhibited SARS-CoV-2 infection in SARS-CoV-2 infected Vero E6 cells (EC_50_ = 22.5 μM) (Wang M. et al., 2020). There are numerous similarities in clinical, pathological, and laboratory findings of moderate to severe SARS-CoV-2 infection and haemophagocytic lymphohistiocytosis. The possibility of etoposide may be considered for treating haemophagocytic lymphohistiocytosis associated with moderately severe or severe forms of SARS-CoV-2 infection (Hamizi et al., 2020). The viroporine channel of COVID-19 can be effectively blocked by amantadine and thus can prevent viral genome release into the cytoplasm. Therefore, amantadine can be used to mitigate the effects of COVID-19 if used in an early stage of infection (Abreu et al., 2020). Niclosamide is another potential option since it can block endocytosis and autophagy of SARS-CoV-2 (Pindiprolu and Pindiprolu, 2020).

Drug repurposing approach by *in silico* studies will give potential clues about the agents which might be helpful to fight this deadly virus. It was found that lopinavir, galidesivir, asunaprevir, CGP42112A, remdesivir, indinavir, ABT450, ritonavir, and methisazone can interact with more than two protein structures of COVID-19. Among these, HIV protease inhibitors exhibited excellent outcomes in docking studies (Shah et al., 2020). Similarly, Wu and associates carried out an analysis by computational methods for discovering therapeutic targets of novel coronavirus as well as predicting potential medicines. The study reports more than 50 natural compounds and more than 50 drugs which may be considered for further studies for the treatment of COVID 19 (Wu et al., 2020). In a study using the drug-target interaction model, atazanavir, remdesivir, efavirenz, ritonavir, dolutegravir, lopinavir, darunavir showed good inhibitory potency, with atazanavir in the first position, followed by remdesivir (Beck et al., 2020). When docking was performed with solvent molecular dynamics on several natural compounds, compounds like 5,7,3’,4’-Tetrahydroxy-2’-(3,3-dimethylallyl) isoflavone, myricitrin, and methyl rosmarinate were observed to be the most promising agents against COVID-19 (ul Qamar et al., 2020). Muralidharan et al. conducted computational studies to understand the synergism of lopinavir, ritonavir, and oseltamivir. They found that the combination of these three drugs resulted in superior binding energy as compared to the individual agents (Muralidharan et al., 2020).

Based on Feline Infectious Peritonitis Strategies, Olsen et al. (2020) suggested the use of nelfinavir and amodiaquine for the treatment of SARS-CoV-2 infection with a potential CNS invasion. *In vitro* study in Vero-E6 cells showed that remdesivir and lopinavir inhibit the replication of SARS-CoV-2 with EC50 at 23.15 and 26.63 μM concentrations, respectively. At the same time, homoharringtonine and emetine have EC50 values of 2.55 and 0.46 μM, respectively, for the inhibition of SARS-CoV-2 replication. In addition, a synergistic effect for the combination of remdesivir and emetine has been also observed (Choy et al., 2020). It may be noted that a 60-year-old immunocompromised female cancer patient on darunavir/cobicistat along with hydroxychloroquine was able to leave the hospital within six days, which shows the potential of darunavir/cobicistat even in immunocompromised patients (Spezzani et al., 2020).

RAC/CDC42-activated kinases (PAK1) are required for the pathogenic process of different kinds of viruses including SARS-CoV-2. Therefore, PAK1 blockers like melatonin, propolis, cicloresonide, some anti-malaria drugs like ivermectin, and ketorolac could act as promising agents against COVID-19 (Maruta and He, 2020). Ivermectin is a broad-spectrum anti-parasitic agent approved by the FDA. An *in vitro* study demonstrated that it can cause a 5000 times decrease in SARS-CoV-2 viral RNA in 48 h and it needs further investigation and clinical trials as a promising therapeutic agent against COVID-19 (Caly et al., 2020). This study received wide attention which finally led USFDA to issue a letter clarifying that the study tested ivermectin neither in humans or animals. The letter also warned the use of ivermectin containing veterinary products in humans (FDA, 2020). The mechanism of action and dose of some potential drugs against COVID-19 are given in Table 4.

**TABLE 4:** The mechanism of action and dose of potential drugs against COVID-19.

Drug	Mechanism of action	Dose
Remdesivir	Inhibition of RNA polymerase	200 mg initial dose after that 100 mg daily (IV) up to 9 days
Chloroquine/Hydroxychloroquine	Modify the transcription process and signaling pathways	400 mg two times on the first day, then 200 mg two times up to 7 days
Umifenovir	Inhibition of membrane fusion	200 mg three times daily maximum up to10 days
Lopinavir/Ritonavir	Protease inhibitor	400/100 mg two times a day for 14 days
Favipiravir	Inhibits viral replication	1600 mg two times in the first day, then 600 mg two times per day up to 6 days
Oseltamivir	Reduce viral replication	75 mg two times daily up to 5 days
Ribavirin	Nucleoside inhibitor	500 mg two or three times daily along with interferon α or lopinavir/ritonavir maximum up to10 days
Metronidazole	Nucleic acid synthesis inhibitor	400 mg two times daily maximum up to 14 days
Baricitinib	Anti-Janus kinase inhibitor	4 mg/day for two weeks
Camostat Mesilate	Block cell entry (Serine protease inhibitor)	200 mg three times daily for 5 days
Darunavir/Cobicistat	Protease inhibitor/inhibitor of cytochrome P450 3A	800 mg/150 mg once daily for 5 days
Thalidomide	Anti-inflammatory, anti-angiogenesis, antifibrotic and immune regulation	100 mg for 14 days
Isotretinoin	Down regulator of ACE-2 receptors also PLpro inhibitor	0.5 mg per kg daily for one month
IFN α	Inhibition of viral replication	atomization: 45μg, two times daily for two weeks or 5 million units or equivalent dose, twice daily not more than 10 days
Fingolimod	Immunology modulator	0.5 mg per day orally for 3 days
Ruxolitinib	JAK1 and JAK2 inhibitor	10 mg two times a day for 14 days with dose reduction or escalation
Tocilizumab	IL-6 blocker	8 mg/kg

## Perspectives

Hopefully, several perspectives and hypotheses are available related to the treatment and prophylaxis against COVID-19. Some of them appear interesting and promising for further consideration and studies. In such a perspective, it is mentioned that ATP has a crucial role in cellular function and cyclic ATP depletion can cause cellular dysfunction including immune cells. ATP-repletion can prevent the “cytokine storm” in COVID-19 and increase the cellular energy to fight against the virus (Taghizadeh-Hesary and Akbari, 2020). Other than the antiviral activity chloroquine/hydroxychloroquine cause the movement of extracellular zinc into intracellular lysosomes and thus hinders the enzyme RNA polymerase which is required for viral replication. Zinc deficiency is commonly seen in geriatrics and also in patients with diabetes, chronic pulmonary disease, cardiovascular diseases, etc. Therefore, zinc supplementation along with chloroquine therapy may reduce the mortality and morbidity rate in COVID-19 (Derwand and Scholz, 2020). Copper is a very important micronutrient required for the functioning of vital immune cells like B cells, helper T cells, natural killer cells, macrophages, etc. Therefore, enhancement of plasma level of copper may boost the immune system and may act as a preventive or therapeutic measure against COVID-19 (Raha et al., 2020). If COVID-19 results in long term cardiopulmonary damage, cardiopulmonary rehabilitation is required; exercise can be considered as a therapy of choice. Since physical movement of many people has become less in this pandemic situation, therefore exercise should be given prime importance. It is proposed that exercise may help in lowering the risk of SARS-CoV-2 infection by minimizing cardiopulmonary sequel in the recovery period (Heffernan and Young Jae, 2020). In a hypothesis, it is postulated that the immunologic effect and enhancement of antibody production by diethylcarbamazine could confer its anti-COVID-19 effect. Diethylcarbamazine is expected to show the immunologic effects by the inhibition of lipoxygenase (LOX) and cyclooxygenase (COX) enzymes (Abeygunasekera and Jayasinghe, 2020). In a similar hypothesis, montelukast is suggested to be useful in restraining the progression of the disease. The anti-inflammatory effect, suppression of oxidative stress, and reduced cytokine production are supposed to facilitate the effect (Fidan and Aydoğdu, 2020). Clinical researches proved the benefit of surfactant treatment in patients with ARDS (Walmrath et al., 1996). Also it was found that early administration of natural lung surfactants can improve the pulmonary function in adult patients with severe respiratory distress syndrome (Mirastschijski et al., 2020). Surfactant based prophylactic management as well as therapy can be considered as another promising strategy (Pramod et al., 2020).

## Conclusion

This review provides an overview of the current treatment strategies, ongoing clinical trials, and potential future options based on published research and registered clinical trials related to the COVID-19. The basics of SARS-CoV-2, the virus, and COVID-19, the disease, are provided. It was noted that none of the present therapies or strategies could be an absolute solution to end this pandemic. The drugs which have a repurposing option are selected based on its activity against RNA viruses like SARS-CoV, MERS-CoV, influenza virus, and Ebola virus. The hope of the successful vaccine, other immunological products, and cell-based therapeutics is still alive. Convalescent plasma therapy is successful in some cases. At present, it could be seen that none of the presently available approaches or drugs was able to stop this pandemic. The results of many clinical trials are yet to be announced. Further, the suggested future potential solutions seem to be promising to end this pandemic.

## Author Contributions

SK: conceptualization, writing – original draft, and funding acquisition. HA: supervision, writing – review and editing. SB-E: writing – review and editing. NA: supervision, writing – review and editing. SM: writing – review and editing. AN: writing – review and editing. PD: writing – review and editing. All authors contributed to the article and approved the submitted version.

## Conflict of Interest

The authors declare that the research was conducted in the absence of any commercial or financial relationships that could be construed as a potential conflict of interest.
